# Vortex vein congestion in the monkey eye: A possible animal model of pachychoroid

**DOI:** 10.1371/journal.pone.0274137

**Published:** 2022-09-01

**Authors:** Hidetaka Matsumoto, Ryo Mukai, Kazuma Saito, Junki Hoshino, Shoji Kishi, Hideo Akiyama

**Affiliations:** Department of Ophthalmology, Gunma University Graduate School of Medicine, Maebashi, Japan; Massachusetts Eye & Ear Infirmary, Harvard Medical School, UNITED STATES

## Abstract

**Purpose:**

To create vortex vein congestion in the monkey eye as a possible pachychoroid model.

**Methods:**

We ligated superotemporal and inferotemporal vortex veins at the surface of the sclera in monkey eyes. Optical coherence tomography (OCT) and indocyanine green angiography (ICGA) were performed before and 2, 7, and 28 days after the vortex vein ligations to investigate changes in vortex vein morphology and alterations in choroidal blood flow.

**Results:**

Before the vortex vein ligations, en face OCT and ICGA images showed well organized vortex veins as well as horizontal and vertical watershed zones. Two days after the vortex vein ligations, dilatation of the superotemporal and inferotemporal vortex veins as well as intervortex venous anastomoses were seen on en face OCT and ICGA images. B-mode OCT images showed choroidal thickening associated with dilatation of the outer choroidal vessels. Moreover, video ICGA revealed choriocapillaris filling delay and pulsatile flow in the dilated vortex veins. At 7 and 28 days after we ligated the vortex veins, these findings were reduced, except for the intervortex venous anastomoses.

**Conclusions:**

We created a monkey model of vortex vein congestion by ligating two vortex veins. This animal model demonstrated pachychoroid-related findings, indicating that vortex vein congestion is involved in the pathogenesis of pachychoroid. However, remodeling of the choroidal drainage route via intervortex venous anastomosis appeared to compensate for the vortex vein congestion created in this model.

## Introduction

Improvements in the performance of optical coherence tomography (OCT) have enabled detailed observation of the choroid, and in 2013 Freund and associates introduced the concept of pachychoroid, describing choroidal thickening associated with dilation of outer choroidal vessels [1]. The results of recent studies have suggested pachychoroid to be associated with the mechanism underlying age-related macular degeneration (AMD) development in Asian populations, accounting for the phenotypic differences between Asian and Caucasian AMD patients [2–4]. Despite various clinical studies having been conducted, the pathogenesis of pachychoroid spectrum diseases has yet to be fully elucidated and optimal treatment strategies for these disorders have yet to be established. Therefore, animal models of pachychoroid are needed.

Several pachychoroid spectrum diseases, including central serous chorioretinopathy (CSC), pachychoroid neovasculopathy (PNV) [5], and polypoidal choroidal vasculopathy (PCV) [6], have been described. Pang et al. demonstrated dilated vortex veins from the distal end to the ampulla in CSC using ultra-widefield indocyanine green angiography (ICGA) [7]. In CSC and PNV, we showed areas of choriocapillaris filling delay in ICGA to overlap extensively with the regions of dilated vortex veins detected on en face OCT images [8, 9]. Moreover, in over 90% of CSC, PNV, and PCV cases, en face OCT images showed anastomosis between superior and inferior vortex veins in the macular area [10–12]. We also detected pulsatile and retrograde flow in the anastomotic vortex veins of CSC, PNV, and PCV eyes [13]. These results raise the possibility that congestion of vortex veins is the main etiology of pachychoroid spectrum diseases [14, 15].

Based on this hypothesis, we created vortex vein congestion in the mouse eye as a possible pachychoroid model by ligating the vortex veins outside the sclera [16]. In this mouse model, choroidal thickening associated with dilatation of choroidal vessels, as seen in pachychoroid spectrum diseases, was observed after vortex vein congestion became established. However, these changes lasted only for several days after the vortex vein ligations. Moreover, it was not possible to assess either intervortex venous anastomosis or macular alterations because the mouse eye lacks the choroidal watershed and macula. Therefore, in the current study, we induced vortex vein congestion in the monkey eye by ligating the vortex veins and then investigated the morphological changes in the vortex veins and alterations in choroidal blood flow.

## Materials and methods

### Animals

All animal procedures were performed in accordance with the ARVO Statement for the use of Animals in Ophthalmic and Vision Research and the National Institutes of Health Guidance for Care and Use of Laboratory animals. The protocol was approved by the Gunma University Animal Care and Experimentation Committee (Permission number: A21-056). Two healthy monkeys (Macaca fuscata) were used in the study. Monkey 1 was a 7.35 kg 10-year-old female and monkey 2 was a 6.95 kg 10-year-old female. Each monkey was individually housed in an indoor cage (0.8m x 0.8m x 1m) on a 12-hr on/12-hr off lighting schedule. The room temperature was kept at 22~23°C with the humidity at 50%. The monkeys were provided ad libitum access to water and food, as well as environmental enrichment (toys). An animal care staff member monitored the health and well-being of the animals daily, checking the consumption of food and conditions of standing and jumping. Before all surgeries and examinations, the monkeys were anesthetized with intramuscular injection of a mixture of ketamine hydrochloride (10 mg/kg) and xylazine hydrochloride (2 mg/kg). Topical ocular surface anesthesia (0.4% oxybuprocaine hydrochloride) was instilled to reduce discomfort. The monkeys were not sacrificed, instead being maintained for later experiments.

### Induction of vortex vein congestion

When all four vortex veins were ligated in the preliminary experiment, hyphema developed postoperatively and the fundus could not be evaluated. In 1973, Hayreh and Baines performed experimental occlusion of monkey vortex veins by cauterization and reported that cauterizing 3–4 vortex veins caused a high rate of hyphema, while cauterizing 1–2 veins resulted in almost no hyphema [17]. Therefore, two vortex veins of the left eye were ligated in this study. After making a 2/3 circumferential incision of the conjunctiva around the limbus, control threads (3–0 SILK BRAID; Alfresa Pharma Corp., Osaka, Japan) were placed under the superior rectus, external rectus, and inferior rectus muscles. The conjunctiva at the superotemporal and inferotemporal quadrants was incised and detached from the sclera. Then, the superotemporal and inferotemporal vortex veins were ligated at the surface of the sclera employing 10–0 nylon surgical suture (MANI, INC., Tochigi, Japan), with the aim of inducing vortex vein congestion (S1 Fig). Finally, the control threads were removed, and the conjunctiva was sutured at the original position with 9–0 silk surgical suture (Alcon, Inc., Tokyo, Japan).

### Optical coherence tomography

OCT was performed before and 2, 7, and 28 days after the vortex vein ligations. Before the examinations, the pupils were dilated with topical drops of a phenylephrine (5%) and tropicamide (0.5%) mixture. We examined the choroidal structure using swept source OCT (Xephilio OCT-S1; Canon Inc., Tokyo, Japan), with 1010–1110 nm near infrared illumination and a scanning speed of 100,000 A-scans/s. To develop en face OCT images, we acquired 3-D volume data of vertical 20 mm (1024 B-scans) x horizontal 23 mm (1024 pixels) scans and vertical 12 mm (1024 B-scans) x horizontal 12 mm (1024 pixels) scans centered on the macula using enhanced depth imaging of swept source OCT. Segmentation of the total choroid was automatically performed using built-in software supported by artificial intelligence, and we set the choroidal thickness as the vertical distance from Bruch’s membrane to the chorioscleral interface. The monkeys were placed on the device, such that the fundus was in focus. OCT images were taken with neither additional contact nor non-contact lenses.

### Indocyanine green angiography

ICGA was performed before and 2, 7, and 28 days after the vortex vein ligations. Before the examinations, the pupils were dilated with topical drops of a phenylephrine (5%) and tropicamide (0.5%) mixture. We injected 12.5mg/1ml of ICG solution into the small saphenous vein of the right leg. We recorded video ICGA for the first 30 seconds after choroidal filling with an angle of 30 degrees centered on the macula using Spectralis HRA + OCT with a light source with a 790 nm emission wavelength (Heidelberg Engineering, Heidelberg, Germany). Then, images were intermittently obtained until 10 minutes after choroidal filling with an angle of 30 degrees centered on the macula using Spectralis HRA + OCT. Ultra-widefield ICGA images were captured 3–5 minutes after choroidal filling by an Optos ultra-wide-field retinal imaging device equipped with a light source with an 802 nm emission wavelength (California; Optos, Dunfermline, Scotland, UK). The monkeys were placed on the devices, such that the fundus was in focus. Video and images of ICGA were taken with neither additional contact nor non-contact lenses.

## Results

**Fig 1:**
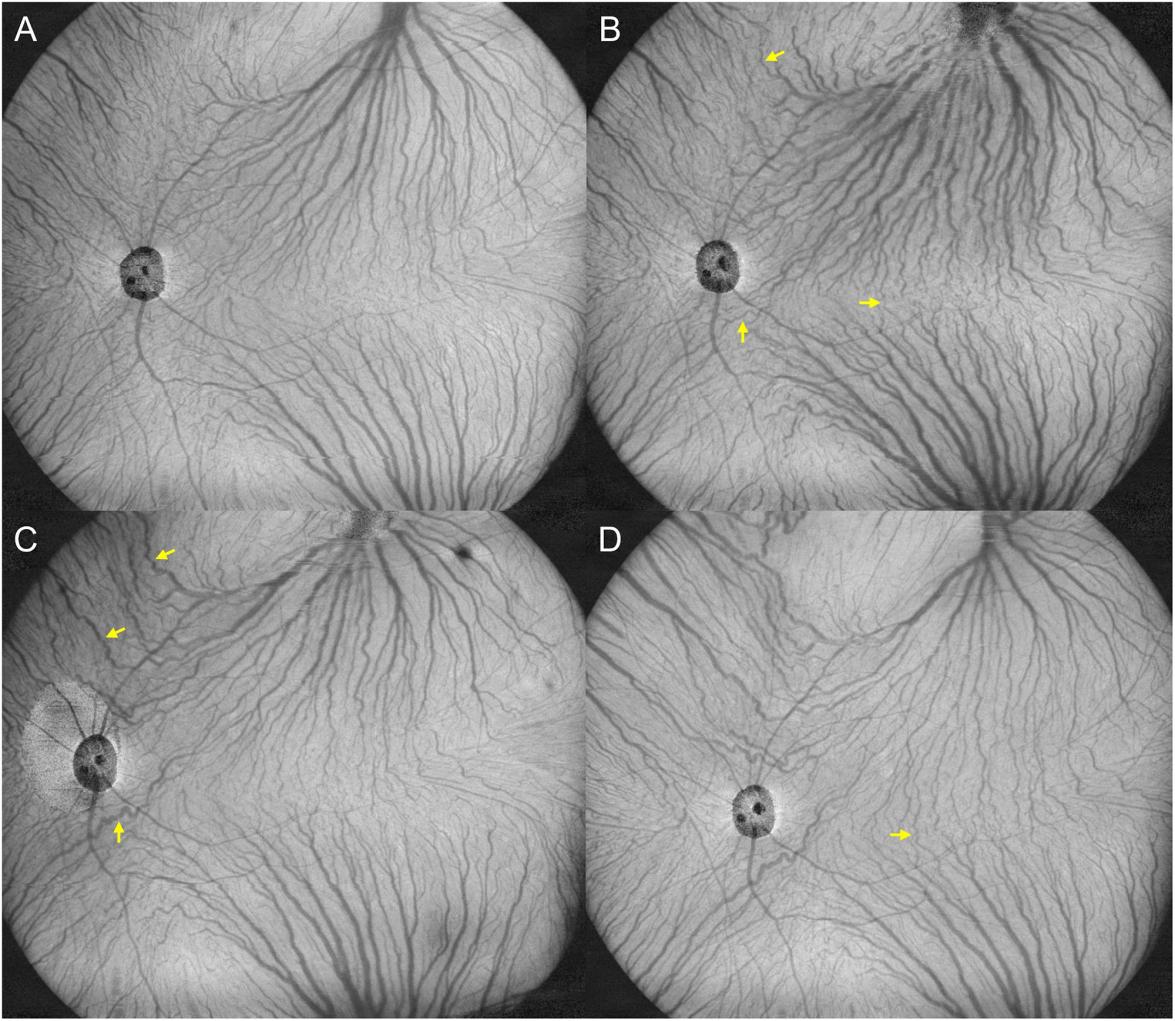
20 x 23 mm en face OCT of the choroid before and 2, 7, and 28 days after ligating the superotemporal and inferotemporal vortex veins in the left eye of monkey 1. (A) Baseline: Vortex veins are well organized and separated into four quadrants by the horizontal and vertical watershed zones. (B) Day 2: Superotemporal and inferotemporal vortex veins are dilated, while several intervortex venous anastomoses have developed across the horizontal and vertical watershed zones (arrows). (C) Day 7: The superotemporal and inferotemporal vortex vein dilations are diminished, while intervortex venous anastomoses across the vertical watershed zone are more dilated than on day 2 (arrows). (D) Day 28: The superotemporal and inferotemporal vortex vein dilatations are reduced, having returned to the baseline level. The anastomotic vessels between the superotemporal and inferotemporal vortex veins are more evident than on days 2 and 7 (arrow).

**Fig 2:**
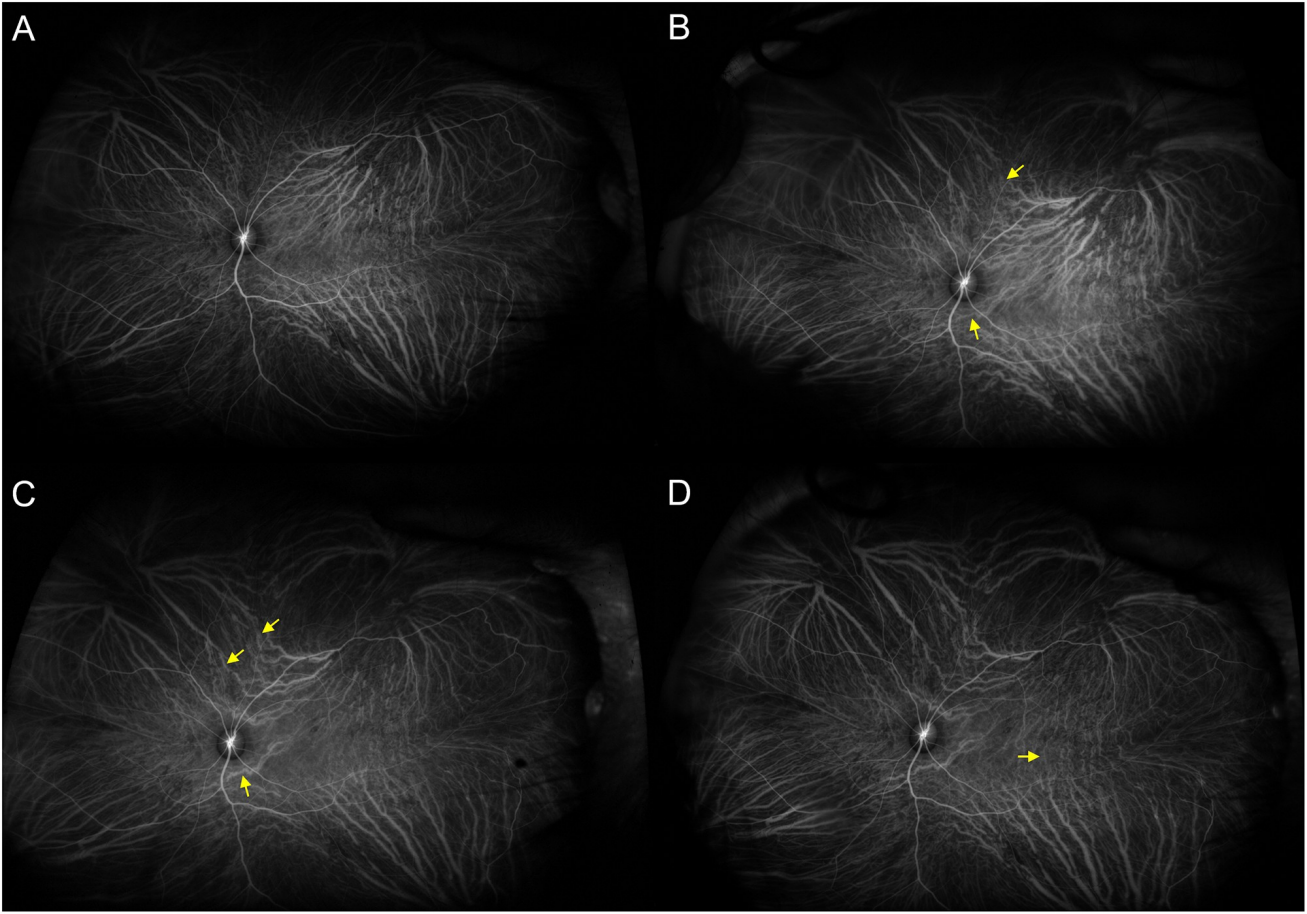
Ultra-widefield ICGA before and 2, 7, and 28 days after ligating the superotemporal and inferotemporal vortex veins in the left eye of monkey 1. (A) Baseline: Vortex veins are well organized and separated into four quadrants by the horizontal and vertical watershed zones. (B) Day 2: Superotemporal and inferotemporal vortex veins are dilated, while limited intervortex venous anastomoses have developed across the vertical watershed zone (arrows). (C) Day 7: The superotemporal and inferotemporal vortex vein dilatations are diminished, while the intervortex venous anastomoses in the vertical watershed zone are more dilated than on day 2 (arrows). (D) Day 28: Intervortex venous anastomoses across the vertical watershed zone persist. In addition, thin anastomotic vessels can be seen between the superotemporal and inferotemporal vortex veins (arrow).

**Fig 3:**
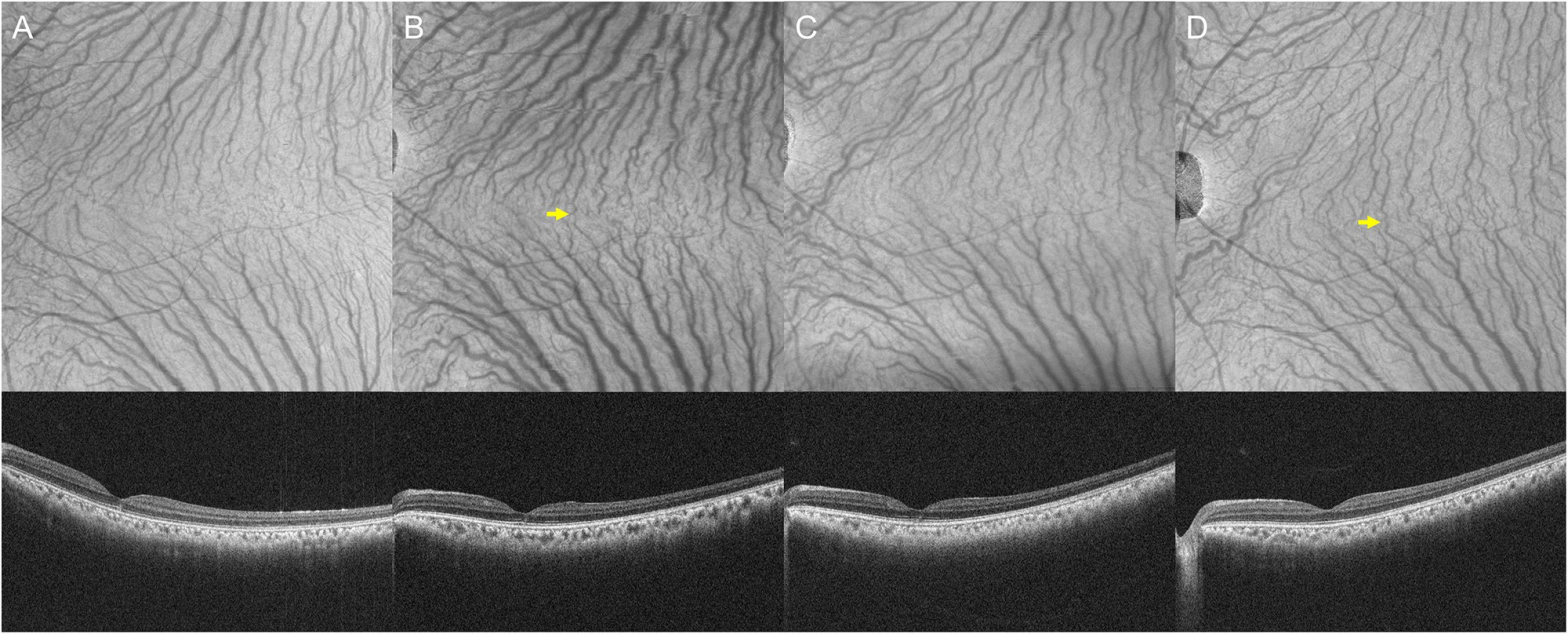
12 x 12 mm en face OCT of the choroid and 12 mm horizontal B-mode OCT through the fovea before and 2, 7, and 28 days after the superotemporal and inferotemporal vortex vein ligations in the left eye of monkey 1. (A) Baseline: En face OCT shows well organized superotemporal and inferotemporal vortex veins separated by the horizontal watershed zone. B-mode OCT shows normal retinal and choroidal structures. The choroidal thickness under the fovea is 153 μm. (B) Day 2: En face OCT reveals the superotemporal and inferotemporal vortex vein dilatations as well as several thin anastomotic vessels between them (arrow). B-mode OCT demonstrates choroidal thickening associated with dilatation of outer choroidal vessels. The choroidal thickness under the fovea is 196 μm. (C) Day 7: En face OCT shows the superotemporal and inferotemporal vortex vein dilatations to be diminished. B-mode OCT reveals choroidal thickening and dilatation of outer choroidal vessels to be reduced. The choroidal thickness under the fovea is 172 μm. (D) Day 28: En face OCT demonstrates that the anastomotic vessels between the superotemporal and inferotemporal vortex veins are more evident than on days 2 and 7 (arrow). B-mode OCT shows several dilated outer choroidal vessels, i.e., the dilated anastomotic vessels between the superotemporal and inferotemporal vortex veins. The choroidal thickness under the fovea is 188 μm.

Quite similar results were obtained from monkeys 1 and 2. Representative monkey 1 is shown in Figs 1–3 and S1-S4 Videos in S1 File. Monkey 2 is shown in S2 and S3 Figs and S5-S8 Videos in S2 File. Before the vortex vein ligations, en face OCT and ICGA images showed well organized vortex veins as well as horizontal and vertical watershed zones. B-mode OCT revealed normal retinal and choroidal structures. Video ICGA showed normal filling of choroidal vessels. Two days after the vortex vein ligations, dilatation of the superotemporal and inferotemporal vortex veins as well as several intervortex venous anastomoses were seen on en face OCT and ICGA images. B-mode OCT images showed choroidal thickening associated with dilatation of the outer choroidal vessels. Moreover, video ICGA revealed choriocapillaris filling delay and pulsatile flow in the dilated vortex veins. Seven days after the vortex vein ligations, superotemporal and inferotemporal vortex vein dilatations were diminished, while anastomotic vortex veins were more dilated than those on day 2 on both en face OCT and ICGA images. The intervortex venous anastomoses were more evident across the vertical than across the horizontal watershed zone. Choroidal thickening and dilatation of the outer choroidal vessels were both diminished on B-mode OCT images. Moreover, choriocapillaris filling delay and pulsatile vortex venous flow detected by video ICGA on day 2 were no longer present on day 7. At 28 days after the vortex vein ligations, the superotemporal and inferotemporal vortex vein dilatations were further reduced, having returned to the baseline level, while the anastomotic vessels among the vortex vein systems were more evident on the en face OCT images. Several dilated outer choroidal vessels, i.e., the dilated anastomotic vessels between the superotemporal and inferotemporal vortex veins, were observed on 12 mm horizontal B-mode OCT images. Video ICGA showed no evidence of choriocapillaris filling delay or pulsatile vortex venous flow on day 28. During the entire study period, there were no indications of choroidal vascular hyperpermeability in the late phase of ICGA.

## Discussion

We endeavored to create vortex vein congestion in the monkey eye as a possible pachychoroid model by ligating vortex veins at the surface of the sclera. This model showed pachychoroid-related findings including dilatation of vortex veins, intervortex venous anastomosis, choroidal thickening, choriocapillaris filling delay, and pulsatile flow in the dilated vortex veins. These findings showed amelioration over time except for the intervortex venous anastomosis. We detected no evidence of choroidal vascular hyperpermeability during the one-month study period.

Takahashi and Kishi reported that new drainage routes of the choroid developed after buckling surgery for retinal detachment [18]. Venovenous anastomoses connected the sector of the occluded vortex veins to that of the intact vortex veins, which compensated for choroidal venous congestion. In the current study, intervortex venous anastomoses developed after the vortex vein ligations. The vortex vein dilatation, choroidal thickening, choriocapillaris filling delay, and pulsatile vortex venous flow were reduced on day 7 as compared to day 2. Therefore, new drainage routes appeared to have developed via intervortex venous anastomoses to compensate for the vortex vein congestion following the vortex vein ligations in our monkey model. Hayreh and Baines evaluated the choroidal circulation in monkey eyes after cauterizing the vortex veins using fluorescein angiography [17]. They reported sluggish and poor choroidal circulation to be observable right after cauterization, but the choroidal circulation had returned to normal on day 7. Chen et al. investigated the choroidal changes after vortex vein ligation in monkeys [19]. They reported that ICGA showed the choroid filling delay in the quadrants of vortex vein occlusion within 1 week. These findings are consistent with our results.

In this study, the intervortex venous anastomoses were more evident across the vertical than across the horizontal watershed zone. We ligated superotemporal and inferotemporal vortex veins in our monkey model. Therefore, the pressure gradient between temporal and nasal vortex veins might have caused the intervortex venous anastomoses across the vertical watershed zone to be more distinct. The intervortex venous anastomoses across the vertical watershed zone had clearly formed on day 7, and thus appeared to compensate for the vortex vein congestion. These anastomotic vessels began to be seen as early as day 2, suggesting that the originally existing tiny anastomotic vessels dilated and became detectable by en face OCT and ultra-widefield ICGA. Dilated anastomotic vessels across the watershed zone are usually detected as dilated outer choroidal vessels on B-mode OCT images. Therefore, we speculate that there might be tiny preexisting anastomotic vessels in Haller’s layer of the choroid. In the current model, the superotemporal vortex veins were extensively connected to the superonasal vortex veins. Thus, the anastomotic vessels between the inferotemporal and superotemporal vortex veins might have developed to reduce the congestion affecting the inferotemporal vortex vein system. Vortex veins can be regarded as tissues rich in plasticity and to thus have a potential compensatory function.

CSC eyes reportedly have thicker sclera than normal control eyes [20]. In pachychoroid spectrum diseases, vortex veins in all quadrants might be chronically strangulated at sites where they penetrate the sclera, thereby producing chronic vortex vein congestion. Therefore, pachychoroid-related findings including vortex vein dilation, intervortex venous anastomosis, choroidal thickening, choriocapillaris filling delay, pulsatile vortex venous flow, and choroidal vascular hyperpermeability might be detectable over a long period of time. In our monkey model, severe acute congestion was induced in the superotemporal and inferotemporal vortex veins. Thus, anastomotic vessels between temporal and nasal vortex veins may compensate for vortex vein congestion. Hence, vortex vein dilation, choroidal thickening, choriocapillaris filling delay, and pulsatile vortex venous flow showed amelioration over time. Moreover, no choroidal vascular hyperpermeability was observed at any time during the one-month study period. Longstanding vortex vein congestion might damage vortex veins or the upstream choriocapillaris, leading to choroidal vascular hyperpermeability.

This study has limitations. Although the pachychoroid phenotype can be attributed to multiple factors, this study focused solely on vortex vein congestion. Most of the data were evaluated subjectively rather than objectively. Vortex vein congestion was ameliorated over time in this monkey model. While we cannot exclude the possibility that the suture effect waned and the vortex veins spontaneously reopened, thereby reducing vortex vein congestion, our meticulous observations indicate this to be unlikely.

In conclusion, vortex vein congestion in the monkey eye, produced by ligating the vortex veins, resulted in vortex vein dilatation, intervortex venous anastomosis, choroidal thickening, choriocapillaris filling delay, and pulsatile vortex venous flow, as seen in pachychoroid spectrum diseases. These results indicate that vortex vein congestion is involved in the pathogenesis of pachychoroid. However, in this model, there appeared to be compensation for vortex vein congestion by remodeling of choroidal drainage routes via intervortex venous anastomosis. We infer that chronic mild vortex vein congestion in all quadrants may be necessary to evaluate the chronic changes associated with pachychoroid.

## Supporting information

S1 Fig Induction of vortex vein congestion. Superotemporal and inferotemporal vortex veins in a monkey eye were ligated at the surface of the sclera employing 10–0 nylon surgical suture, with the aim of inducing vortex vein congestion.
(TIF)

S2 Fig 20 x 23 mm en face OCT of the choroid before and 2, 7, and 28 days after ligating the superotemporal and inferotemporal vortex veins in the left eye of monkey 2. (A) Baseline: Vortex veins are well organized and separated into four quadrants by the horizontal and vertical watershed zones. (B) Day 2: Superotemporal and inferotemporal vortex veins are dilated, while several tiny intervortex venous anastomoses have developed across the vertical and horizontal watershed zones (arrows). (C) Day 7: The superotemporal and inferotemporal vortex vein dilations are diminished, while the intervortex venous anastomoses are more dilated than on day 2 (arrows). (D) Day 28: The superotemporal and inferotemporal vortex vein dilatations are reduced, having returned to the baseline level, while the anastomotic vessels persist.
(TIF)

S3 Fig Ultra-widefield ICGA before and 2, 7, and 28 days after ligating the superotemporal and inferotemporal vortex veins in the left eye of monkey 2. (A) Baseline: Vortex veins are well organized and separated into four quadrants by the horizontal and vertical watershed zones. (B) Day 2: Superotemporal and inferotemporal vortex veins are dilated, while limited intervortex venous anastomoses have developed across the vertical watershed zone (arrow). (C) Day 7: The superotemporal and inferotemporal vortex vein dilatations are diminished, while the intervortex venous anastomoses in the vertical watershed zone are more dilated than on day 2 (arrows). In addition, thin anastomotic vessels can be seen between the superotemporal and inferotemporal vortex veins (arrows). (D) Day 28: Intervortex venous anastomoses across the vertical and horizontal watershed zones persist.
(TIF)

S1 File Video ICGA before and 2, 7, and 28 days after superotemporal and inferotemporal vortex vein ligations in the left eye of monkey 1. S1 Video. Video ICGA at baseline shows normal filling of choroidal vessels. S2 Video. Video ICGA on day 2 demonstrates choriocapillaris filling delay and pulsatile flow in the dilated vortex veins. S3 Video. Video ICGA on day 7 confirms the absence of both choriocapillaris filling delay and pulsatile vortex venous flow. S4 Video. Video ICGA on day 28 shows normal filling of choroidal vessels.
(ZIP)

S2 File Video ICGA before and 2, 7, and 28 days after superotemporal and inferotemporal vortex vein ligations in the left eye of monkey 2. S5 Video. Video ICGA at baseline shows normal filling of choroidal vessels. S6 Video. Video ICGA on day 2 demonstrates focal choriocapillaris filling delay and pulsatile flow in the dilated vortex veins. S7 Video. Video ICGA on day 7 confirms the absence of both choriocapillaris filling delay and pulsatile vortex venous flow. S8 Video. Video ICGA on day 28 shows normal filling of choroidal vessels.
(ZIP)
