# Supplementary figures and images for: Vortex vein congestion in the monkey eye: A possible animal model of pachychoroid
Source: PLoS One. 2022 Sep 1;17(9):e0274137. doi: 10.1371/journal.pone.0274137 (PMC9436071; doi:10.1371/journal.pone.0274137)

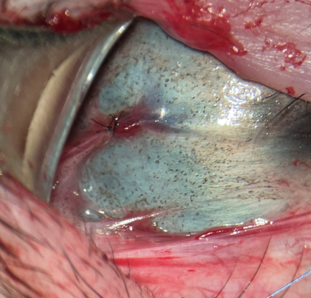

Supplement: S1 Fig — Superotemporal and inferotemporal vortex veins in a monkey eye were ligated at the surface of the sclera employing 10–0 nylon surgical suture, with the aim of inducing vortex vein congestion. (TIF) [file pone.0274137.s001.tif]

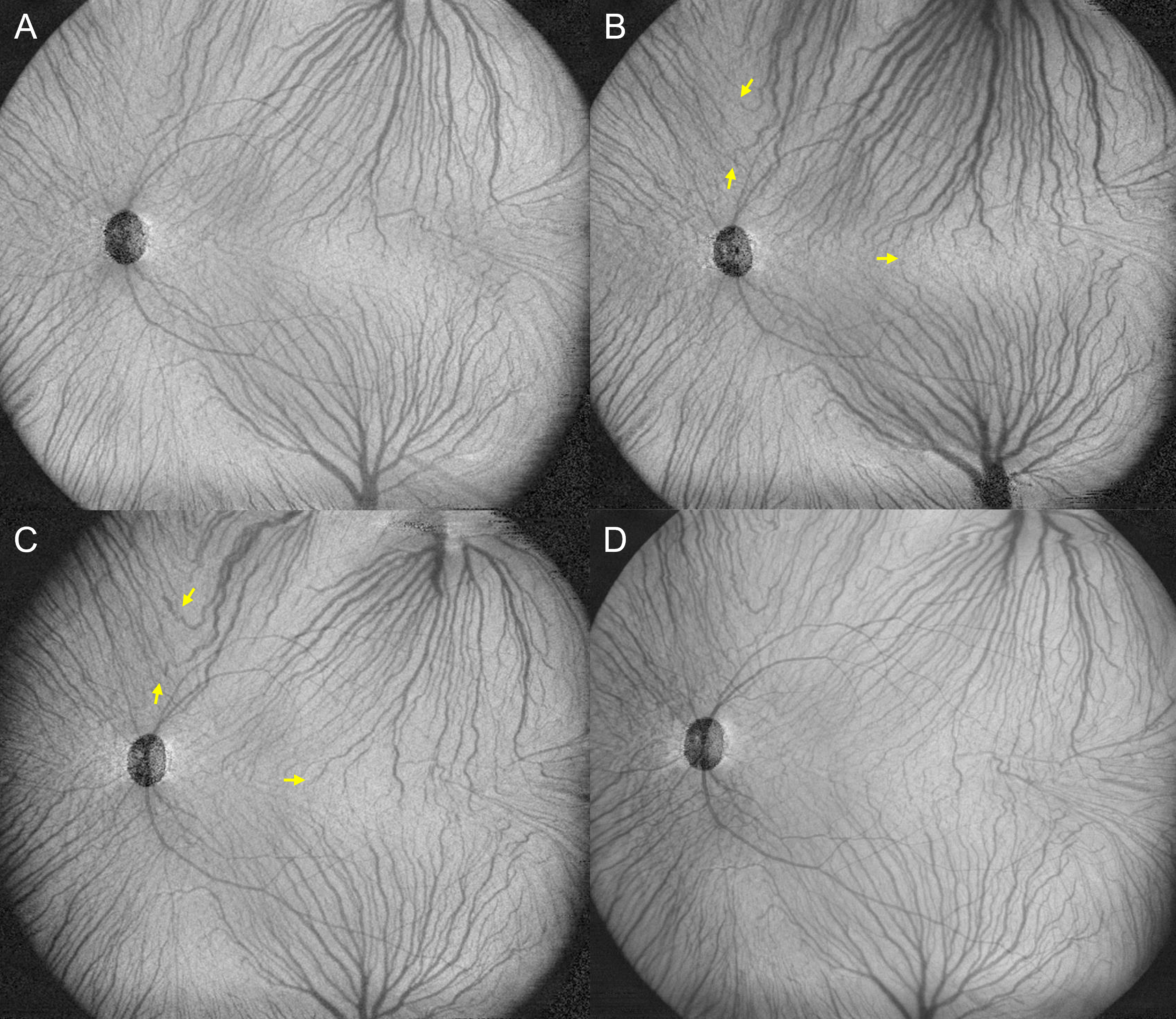

Supplement: S2 Fig — (A) Baseline: Vortex veins are well organized and separated into four quadrants by the horizontal and vertical watershed zones. (B) Day 2: Superotemporal and inferotemporal vortex veins are dilated, while several tiny intervortex venous anastomoses have developed across the vertical and horizontal watershed zones (arrows). (C) Day 7: The superotemporal and inferotemporal vortex vein dilations are diminished, while the intervortex venous anastomoses are more dilated than on day 2 (arrows). (D) Day 28: The superotemporal and inferotemporal vortex vein dilatations are reduced, having returned to the baseline level, while the anastomotic vessels persist. (TIF) [file pone.0274137.s002.tif]

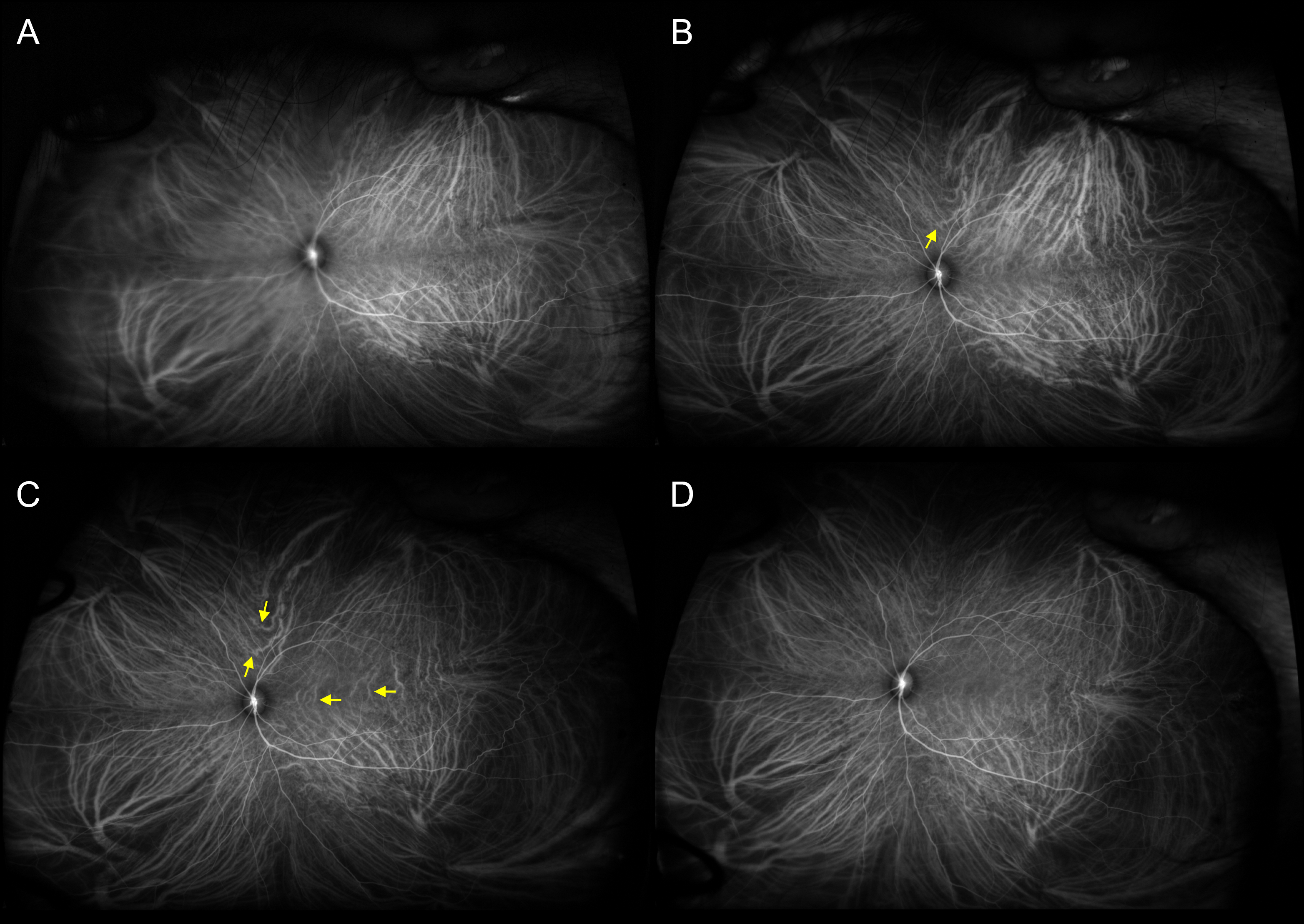

Supplement: S3 Fig — (A) Baseline: Vortex veins are well organized and separated into four quadrants by the horizontal and vertical watershed zones. (B) Day 2: Superotemporal and inferotemporal vortex veins are dilated, while limited intervortex venous anastomoses have developed across the vertical watershed zone (arrow). (C) Day 7: The superotemporal and inferotemporal vortex vein dilatations are diminished, while the intervortex venous anastomoses in the vertical watershed zone are more dilated than on day 2 (arrows). In addition, thin anastomotic vessels can be seen between the superotemporal and inferotemporal vortex veins (arrows). (D) Day 28: Intervortex venous anastomoses across the vertical and horizontal watershed zones persist. (TIF) [file pone.0274137.s003.tif]
